# Ischaemic stroke in mice induces lung inflammation but not acute lung injury

**DOI:** 10.1038/s41598-019-40392-1

**Published:** 2019-03-06

**Authors:** Victoria Austin, Jacqueline M. Ku, Alyson A Miller, Ross Vlahos

**Affiliations:** 1 School of Health and Biomedical Sciences, RMIT University, Bundoora, Victoria, 3083 Australia; 2 Institute of Cardiovascular & Medical Sciences, Glasgow Cardiovascular Research Centre, University of Glasgow, Glasgow, Scotland United Kingdom

## Abstract

Stroke is a major cause of death worldwide and ischemic stroke is the most common subtype accounting for approximately 80% of all cases. Pulmonary complications occur in the first few days to weeks following ischemic stroke and are a major contributor to morbidity and mortality. Acute lung injury (ALI) occurs in up to 30% of patients with subarachnoid haemorrhage but the incidence of ALI after ischemic stroke is unclear. As ischemic stroke is the most common subtype of stroke, it is important to understand the development of ALI following the initial ischemic injury to the brain. Therefore, this study investigated whether focal ischemic stroke causes lung inflammation and ALI in mice. Ischemic stroke caused a significant increase in bronchoalveolar lavage fluid (BALF) macrophages and neutrophils and whole lung tissue proinflammatory IL-1β mRNA expression but this did not translate into histologically evident ALI. Thus, it appears that lung inflammation, but not ALI, occurs after experimental ischemic stroke in mice. This has significant implications for organ donors as the lungs from patient’s dying of ischemic stroke are not severely damaged and could thus be used for transplantation in people awaiting this life-saving therapy.

## Introduction

Stroke is currently the second-leading global cause of death behind heart disease, accounting for approximately 12% of total deaths worldwide^1^. Ischemic strokes are the most common subtype of stroke, accounting for approximately 80% of all cases^2^. Medical complications are common after stroke, and these are a major contributor to morbidity and mortality^3,4^. Pulmonary complications such as pneumonia, acute lung injury (ALI) and neurogenic pulmonary oedema (NPO) frequently occur in the first few weeks following a stroke^5–8^. ALI is commonly associated with various forms of brain injury and is estimated to occur in 5–30% of patients with subarachnoid haemorrhage, a subtype of stroke^6,8,9^. The incidence of ALI after ischaemic stroke is unclear; however one study reported acute respiratory distress syndrome (ARDS, a more severe form of ALI) in 4% of ischaemic stroke patients^10^. As ischemic strokes are the most common subtype of stroke, it is important to understand the development of ALI following the initial injury to the brain.

ALI has an estimated mortality rate of 40%, while ARDS has a mortality rate closer to 46%. In patients hospitalised with traumatic brain injury, the risk of mortality increases by approximately 20% in individuals with ALI/ARDS^11^, and severity of brain injury is a risk factor for development of ALI^12^. Additionally, ALI and ARDS can lead to significant physical and neurocognitive impairment, likely due to hypoxia. Ischemic stroke is associated with long-term physical and cognitive impairment; thus, it is reasonable to assume that ALI/ARDS could worsen these impairments. ALI makes lungs from potential organ donors unsuitable for transplant. It is estimated that 42% of lung donors die of stroke and therefore it is necessary to understand the occurrence of ALI in the context of ischemic stroke. This is critical because ALI would restrict the total number of lungs available from stroke patients for the many patients awaiting this life-saving therapy. Additionally, stroke-induced ALI may worsen lung injury caused by mechanical ventilation^13,14^.

ALI is a clinical syndrome characterised by an acute inflammatory process in the lung tissue and airways, leading to an acute onset of severe hypoxemia^15^. This influx of inflammation is due to the loss of barrier function of the lung epithelial cells and the pulmonary capillary endothelial cells^16^. Neutrophils adhere to the damaged endothelium and migrate into the lung airspaces. Cytokines such as IL-1, IL-6 IL-8, IL-10 and TNF-a are secreted by alveolar macrophages, which stimulates chemotaxis and activation of neutrophils^17^. Neutrophilic inflammation causes injury to the lung endothelium, in turn increasing capillary permeability^18^. This leads to an influx of protein-rich fluid into the airspaces, and ultimately pulmonary oedema. Diffuse alveolar damage and impaired gas exchange results from the culmination of these processes.

It is well established that ischemic stroke causes brain inflammation but whether this cerebrovascular damage translates into lung inflammation is not well explored. A recent study in rats showed that focal ischemic stroke is associated with brain-lung crosstalk, leading to increased pulmonary damage and inflammation, as well as reduced alveolar macrophage phagocytic capability, which seems to be promoted by systemic inflammation^19^. *Streptococcus pneumoniae* infection increased the levels of the inflammatory cytokines TNF-α, IL-6 and IL-1β after cerebral ischemia (middle cerebral artery occlusion) in the lung compared to uninfected mice^20^. It has also been shown that experimental stroke in mice induces a peripheral inflammatory response that peaks 4 h after stroke and precedes the peak in brain inflammation 24 h after stroke^21^. A recent study has shown that ALI appears to occur within 24 h of ischemic stroke^22^. These findings suggest that an investigation of lung inflammation and injury induced by stroke would need to draw a focus on the hours shortly after a stroke.

Therefore, the aim of this study was to determine whether ischemic stroke causes lung inflammation and ALI in mice, in the period shortly after stroke (6 h), and at times (24 and 72 h following stroke) when the stroke is more developed. As severity of brain injury is a risk factor for the development of ALI in the context of traumatic brain injury^12^, we also investigated whether there was lung inflammation and injury after experimental stroke using a longer ischemic period to model a more severe stroke. We hypothesised that lung inflammation and ALI would occur after experimental stroke, and it would be highest at earlier time-points and after a more severe stroke.

## Materials and Methods

### Mice

All experiments were conducted in accordance with the Australia Code of Practice for the Care of Experimental Animals, the ARRIVE Guidelines and with RMIT University Animal Ethics Committee approval (AEC numbers 1349 and 1532). Male 7–12-week-old C57BL/6 mice (n = 89 total) were obtained from the Animal Resources Centre Pty. Ltd (Perth, Australia). Animals were housed at 20 °C on a 12 h light/dark cycle and had access to water and standard chow ad libitum. In all, 22 mice were excluded from the study which occurred when, during the surgical procedure to induce focal cerebral ischemia-reperfusion: (1) there was an inadequate reduction (<70%) in regional cerebral blood flow (rCBF) during the ischemic period or inadequate (>80%) increase within the first 10 minutes of reperfusion (n = 9); (2) technical or anaesthesia complications arose during surgery (n = 1); (3) they died prior to the end of the reperfusion period (n = 5); or (4) they had to be humanely killed prior to the end of reperfusion (according to clinical severity score; n = 7).

### Focal cerebral ischemia and reperfusion

Mice were anaesthetised with a mixture of ketamine (150 mg/kg, i.p.) and xylazine (10 mg/kg, i.p.). Body temperature was maintained at 37 °C with a heat lamp throughout the procedure and until mice regained consciousness. Focal cerebral ischemia and reperfusion was performed on mice by transient intraluminal filament-induced middle cerebral artery occlusion (tMCAo) as previously described^23–25^. Cerebral ischemia was maintained for either 50 min or 60 min. rCBF in the area of the cortex supplied by the middle cerebral artery (MCA) (~2 mm posterior and 5 mm lateral to bregma) was monitored in all stroke mice and recorded prior to the induction of cerebral ischemia, during cerebral ischemia and for the first 10 min of reperfusion. For sham surgeries, the right external carotid artery and common carotid artery were visualised, but the filament was not inserted. After mice had recovered from anaesthesia, they were housed in individual cages. Mice were monitored hourly for a minimum of 8 h post-surgery and the following morning using our monitoring protocol and clinical signs severity scoring system (approved by our ethics committee). At the end of the experiment (6 hours, 24 hours, or 72 hours post-surgery), mice were killed with an overdose of isoflurane followed by decapitation.

### Neurological scoring and functional impairment test

Neurological assessment was performed 24 and 72 h after either sham or stroke surgery using a five-point scoring system: 0 = normal motor function; 1 = flexion of torso and contralateral forelimb when lifted by the tail; 2 = circling to the contralateral side when held by the tail on a flat surface with normal posture at rest; 3 = leaning on the contralateral side at rest; 4 = no spontaneous movement at rest or uncontrolled circling. A hanging wire test was performed at 24 h and 72 h after sham or stroke surgery to assess motor impairment, as previously described^23^. Briefly, mice were suspended by their forelimbs from a wire 30 cm above a padded surface for up to 60 s and the average hanging time (i.e. latency to fall) of 3 trials with 5 min rest in between was recorded. A score of zero was assigned to those mice that fell immediately and a score of 60 was assigned to animals that did not fall. Neurological scoring and functional impairment tests were not performed 6 h after sham or stroke surgery, as mice have not fully recovered from the effects of anaesthesia at this time-point.

### Quantification of cerebral infarct and oedema volumes

Cerebral infarct and oedema volumes were evaluated at 6 h, 24 h or 72 h after stroke surgery, as previously described^23^. Briefly, brains were coronally sectioned (30 µm thickness; 420 µm apart) and thaw-mounted onto 0.1% poly-L-lysine coated slides. Tissue-mounted slides were subsequently stained with 0.1% thionin to delineate the infarct. Thionin-stained sections were then imaged with an Olympus VS120 Slide Scanner (Olympus). Total infarct volume was then quantified using ImageJ analysis software, correcting for brain oedema, as previously described^23,25^.

### Bronchoalveolar lavage and differential cell counts

Lungs from each terminally anaesthetised mouse were lavaged *in situ* with a 400 µl aliquot, followed by three 300 µl aliquots of PBS as previously described^26–28^. In total up to 1 ml of bronchoalvealor lavage fluid (BALF) was retrieved per mouse. The total number of viable cells in the BALF was determined, cytospins prepared and cells differentiated by standard morphological criteria. The total number of viable cells in the BALF was determined using the fluorophores ethidium bromide and acridine orange (AO/EB), on a Nikon Eclipse E600 (Nikon Instruments, USA). Cytospins were prepared using 100 μl of BALF spun at 400 rpm for 10 min using a Cytospin 3 (Shandon, UK). Cytospin preparations were then stained with DiffQuik (Dade Baxter, Australia), and 500 cells per slide were counted and differentiated into macrophages, neutrophils and lymphocytes using standard morphological criteria. The remaining BALF was centrifuged and the supernatant stored at −80 °C until required for further analysis. Whole lungs were cleared of blood via right ventricular perfusion of the heart with 5 ml of PBS, rapidly excised en bloc, snap-frozen in liquid nitrogen and stored at −80 °C until required.

### RNA extraction and qPCR

Lungs from individual mice were crushed to a fine powder in liquid nitrogen using a mortar and pestle, and subsequently homogenised by passing 5 times through a 21 G needle with a 1 ml syringe. Total RNA was extracted from 15 mg lung samples using an RNeasy Plus kit (QIAGEN, Australia), according to the manufacturer’s instructions. RNA yield and purity were quantified using a nanodrop (ND-1000, Biolab). Total RNA from lung samples were reverse transcribed to cDNA (Applied Biosystems High Capacity RNA-to-cDNA Kit, USA). Quantitative polymerase chain reaction (qPCR) was then performed using mouse-specific TaqMan® Gene Expression Assays (Applied Biosystems, USA), on an ABI 7900HT Sequence Detection System. Samples were assayed in triplicate and negative reverse-transcriptase controls were included. Fold change was determined relative to the sham control group, after standardising to GAPDH (housekeeping gene), using the standard 2^(-ΔΔCT)^ method as previously published^26–29^.

### Lung histology

At the end of the experiments, the lung tissues were fixed with 4% paraformaldehyde for 24 h and embedded in paraffin. After deparaffinisation and dehydration, the lungs were cut into 5-μm sections and stained with haematoxylin and eosin. Histology was performed as previously described^27^. Briefly, lungs were removed from the thorax and immersed in 4% formaldehyde for a minimum period of 24 h. After fixation of the lung tissue and processing in paraffin wax, sections (5 µm thick) were cut longitudinally through the left and right lung so as to include all lobes. Sections were stained with hematoxylin and eosin for general histopathology. Histological evidence of ALI was assessed by the presence of neutrophils in the alveolar or interstitial space; the formation of hyaline membranes; presence of proteinaceous debris; and thickening of the alveolar walls^30^.

### Data analysis

All results are presented as mean ± standard error of the mean (SEM); *n* represents the number of mice. Statistical comparisons between treatment groups were performed using either Student’s unpaired t test or one-way ANOVA with Sidak’s multiple comparisons post-hoc test. Mann–Whitney U test was used for non-parametric data. All statistical analyses were performed using GraphPad Prism 6 for Windows (Version 6.07, La Jolla, CA, USA). Probability levels less than 0.05 (P < 0.05) were taken to indicate statistical significance.

## Results

### Functional and neurological outcomes of stroke (50 min occlusion)

**Figure 1:**
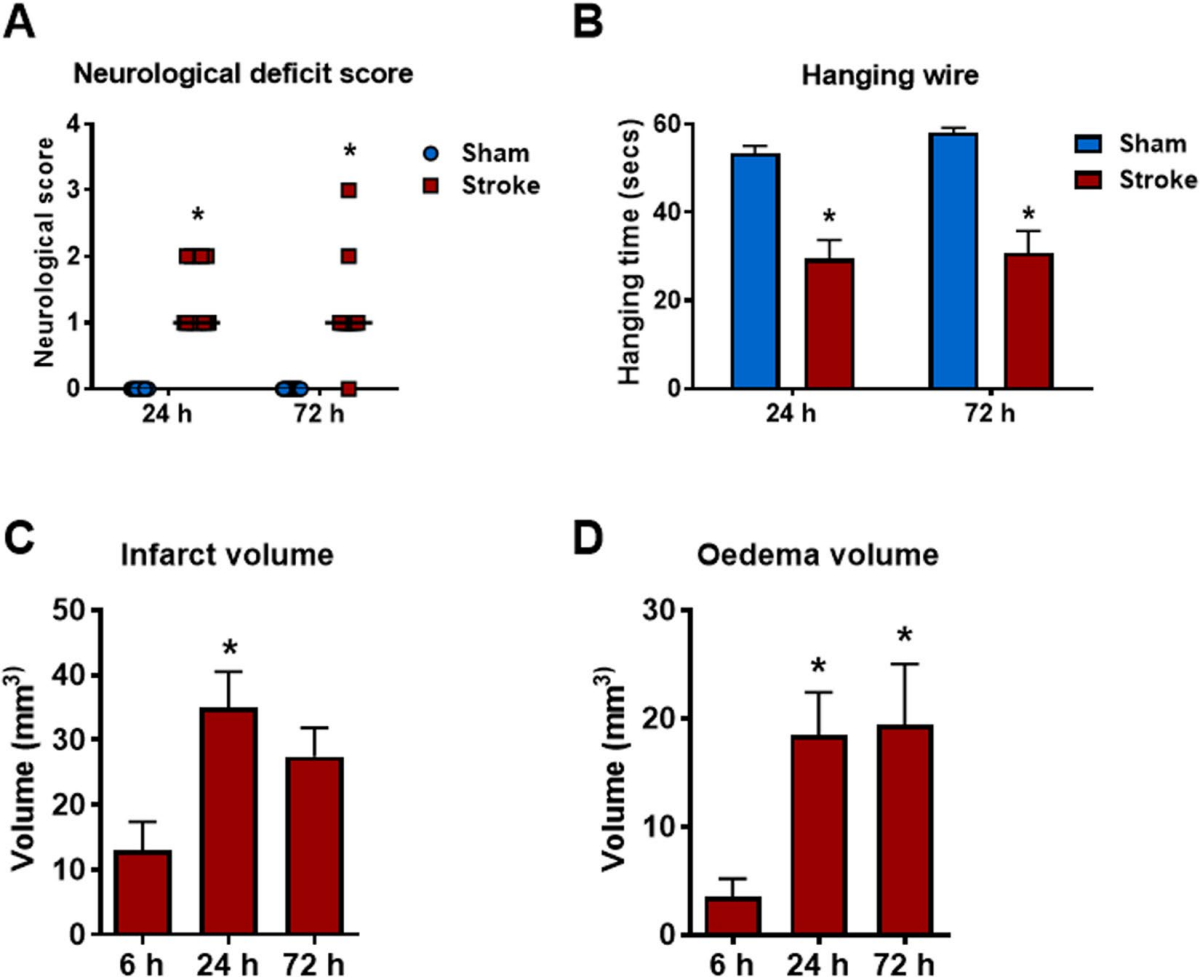
Stroke outcomes at 6 h, 24 h and 72 h after 50 min ischaemia. Neurological deficit scores and hanging wire tests (**A**,**B**; n = 10–15), infarct and oedema volumes (**C**,**D**; n = 6–11). Data are expressed as mean ± SEM. Neurological deficit score data expressed as median (*P < 0.05 vs sham, Mann-Whitney test). Hanging wire test was analysed by two-way ANOVA (*P < 0.05 vs sham). Infarct and oedema data was analysed by one-way ANOVA followed by Sidak *post-hoc* test (*P < 0.05).

Stroke mice displayed significant neurological impairment compared to sham mice at 24 and 72 h post-stroke, however no difference in neurological impairment was observed when comparing stroke groups at 24 h and 72 h (Fig. 1A). Foregrip strength, as assessed by latency to fall in the hanging wire test, was significantly less in stroke mice at 24 and 72 h when compared to sham-operated mice (Fig. 1B). However, no differences in hanging grip times were observed between the 24 and 72 h stroke groups. There were detectable infarct and oedema volumes at 6, 24 and 72 h post-stroke (Fig. 1C,D). Infarct and oedema volumes were greater at 24 and 72 h when compared to 6 h post-stroke. Neurological impairment and foregrip strength were not assessed at 6 h, as this time-point is too early to see changes in functional impairment and mice have not fully recovered from the effects of anaesthesia. There were no detectable infarcts or oedema in sham-operated mice (data not shown).

### Effect of stroke on BALF cellularity and lung weight, structure and proinflammatory gene expression

**Figure 2:**
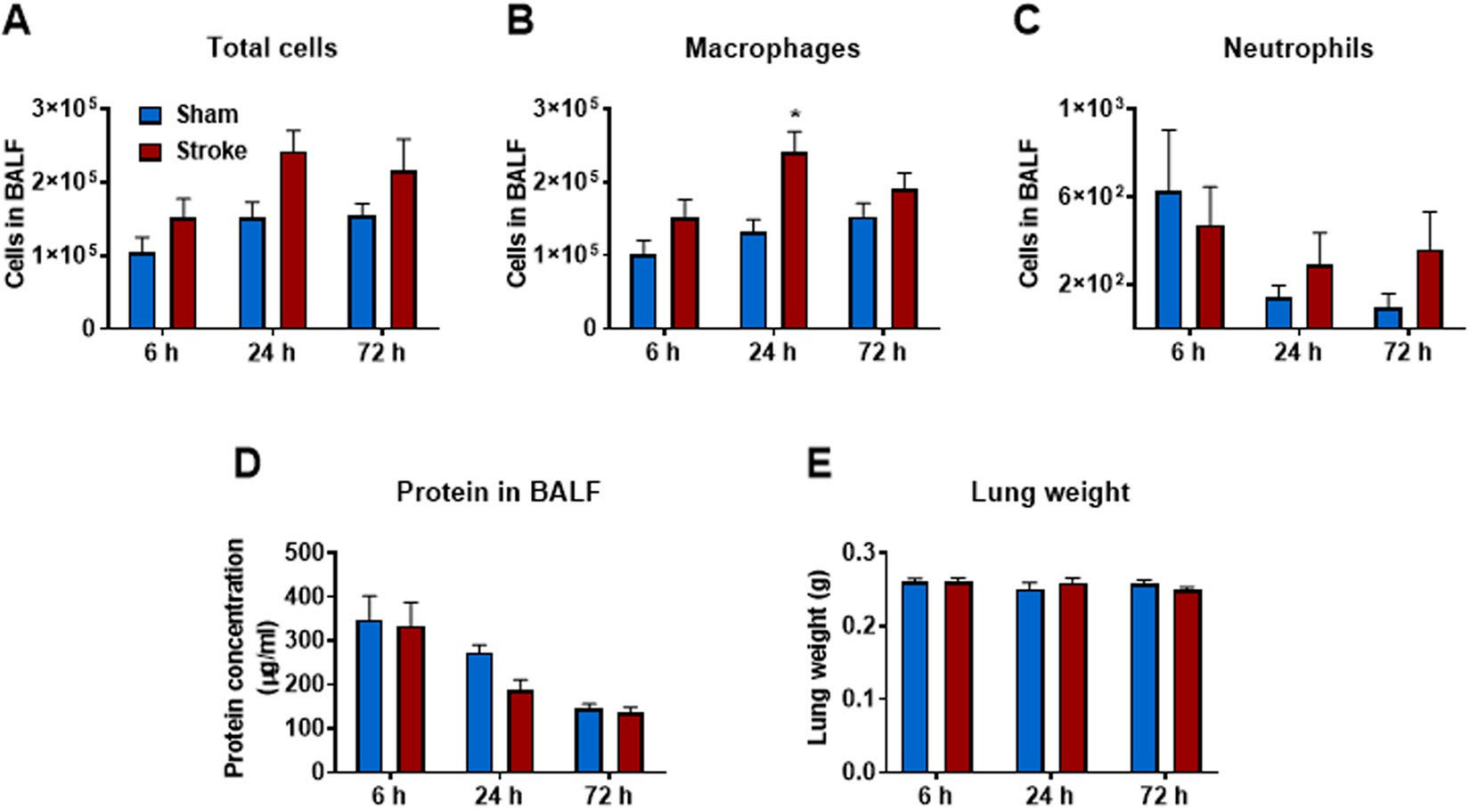
Effect of 50 min ischaemia on inflammatory cell counts in BALF, lung weight and BALF protein concentration. BALF cellularity is shown as (**A**) the total number of cells, (**B**) macrophages and (**C**) neutrophils (n = 10–15). Protein concentration in BALF (**D**) and lung weight (**E**) data are also shown (n = 9–13). Data are expressed as mean ± SEM. Two-way ANOVA followed by Sidak *post-hoc* test (*P < 0.05).

**Figure 3:**
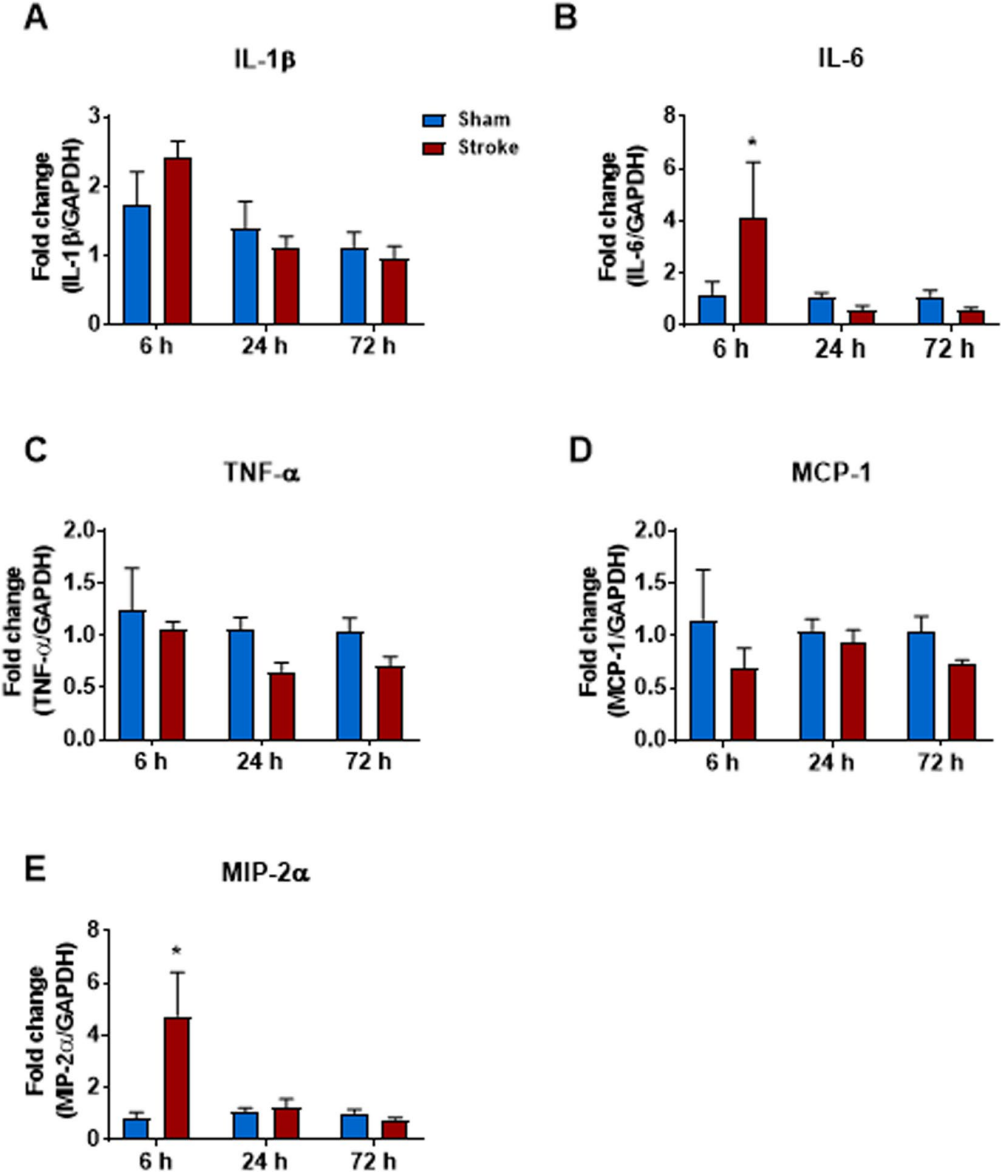
Effect of 50 min ischaemia on mRNA expression of proinflammatory cytokines and chemokines in whole lung tissue. Data are expressed as mean ± SEM. Two-way ANOVA followed by Sidak *post-hoc* test (*P < 0.05; n = 6–12).

**Figure 4:**
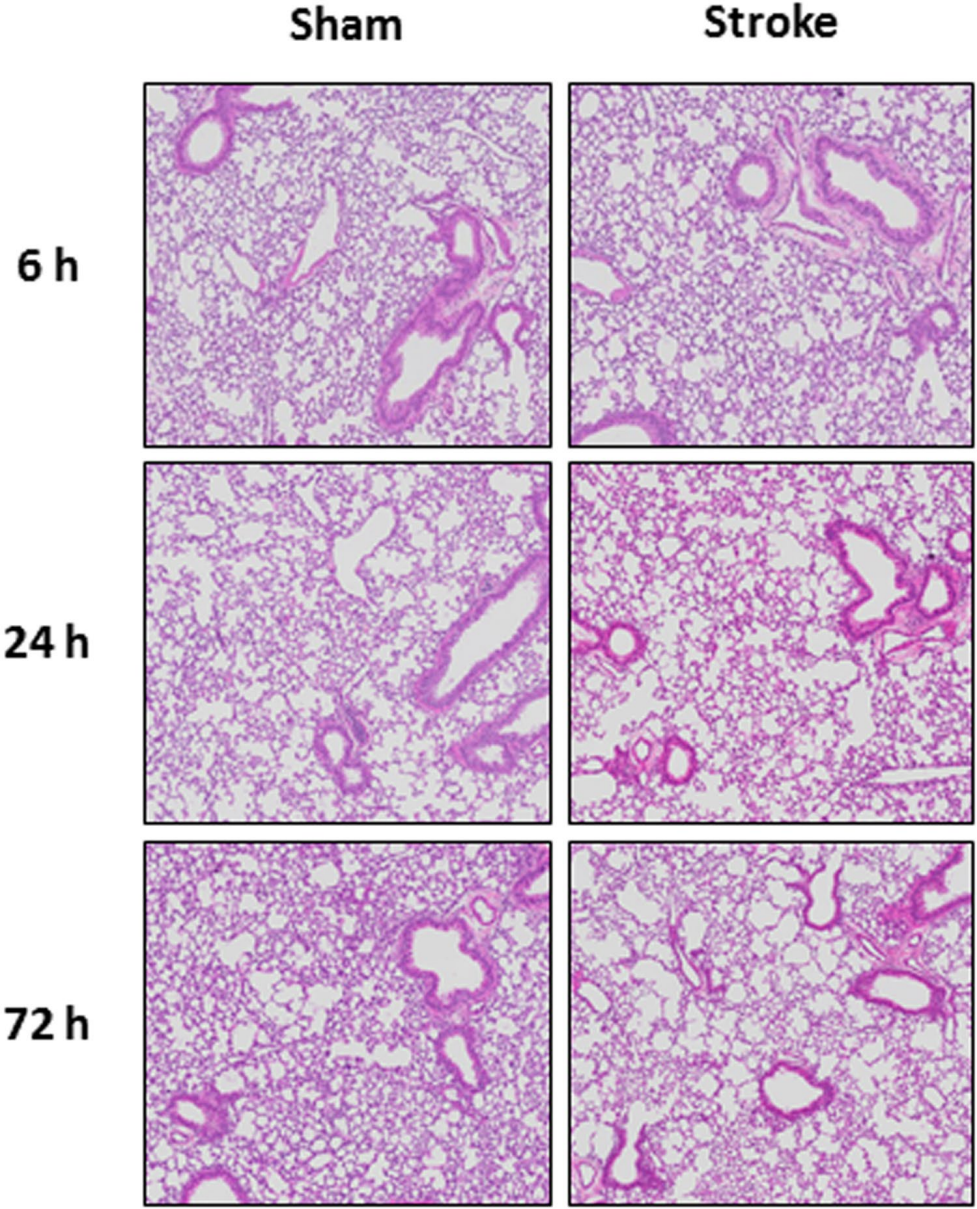
Hematoxylin and eosin stained lung tissue section from mice 6, 24 and 72 h post stroke (50 min ischaemia) or sham surgery.

There was an increase in total cells in BALF at 6, 24 and 72 h when compared to sham-operated mice although this was not statistically significant (Fig. 2A). Similarly, there were no statistical differences in the number of neutrophils at 6, 24 or 72 h post-stroke (Fig. 2C). Although there was no difference in the number of macrophages 6 and 72 h post-stroke, there was however a significant increase in the number of macrophages at 24 h post-stroke (Fig. 2B). Stroke did not affect lung weights or BALF protein content at any of the time-points investigated (Fig. 2D,E). Whole lung gene expression of IL-1β, TNF-α and MCP-1 was not altered at any of the time-points post stroke, but there was an increase in IL-6 and MIP-2α at the 6 h time-point (Fig. 3). No evidence of lung injury was observed following stroke at any of the time points investigated as assessed by histological examination of lung tissue (Fig. 4).

### Functional and neurological outcomes of stroke (60 min occlusion)

**Figure 5:**
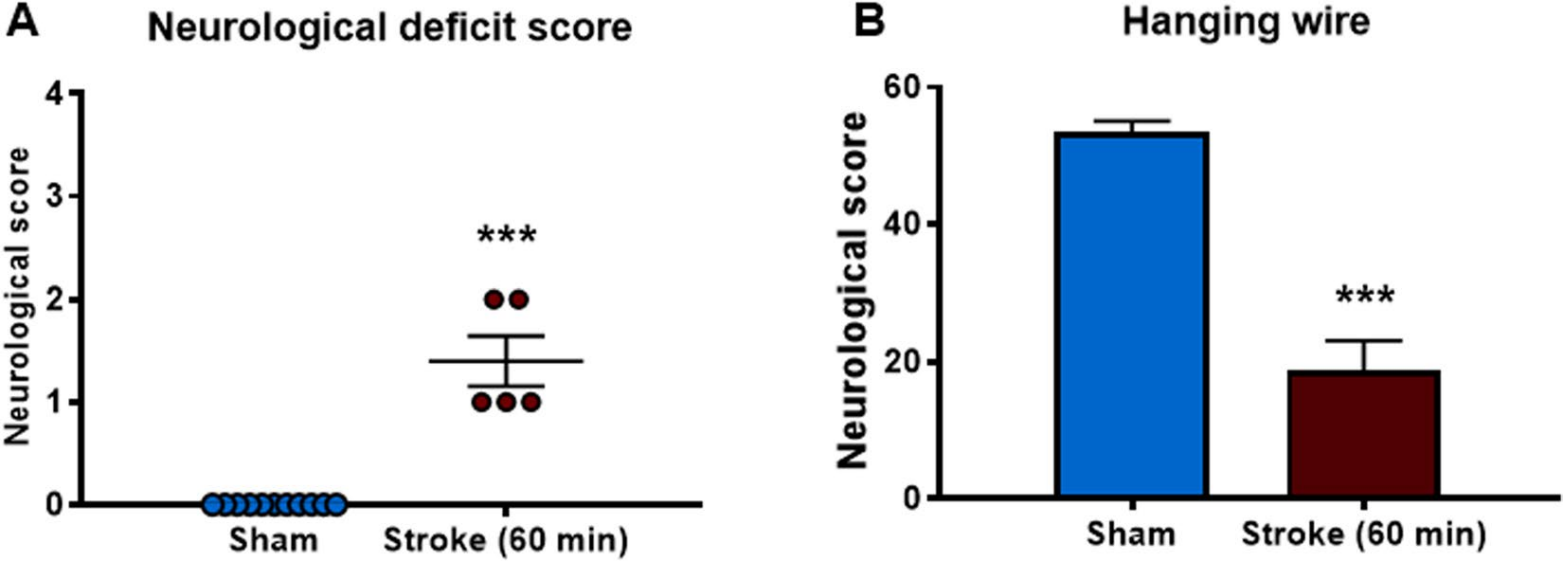
Stroke outcomes at 24 h after 60 min ischaemia. Neurological deficit scores (**A**) expressed as median (***P < 0.0005 vs sham, Mann-Whitney test; n = 5–11). Hanging wire test (**B**) expressed as mean ± SEM (***P < 0.0001 vs sham, Student’s t-test; n = 5–11).

Three-time points were studied in the 50 min occlusion study to determine the kinetics of the inflammatory response in the lung. From this study we found that there were differences in BALF inflammation (macrophages) only at the 24-hour time-point. Hence, we chose to only investigate the 24-hour time-point in the 60 min occlusion study as this would also limit the number of animals used for the study. Stroke mice displayed significant neurological impairment compared to sham mice 24 h post-stroke (Fig. 5A). Foregrip strength was significantly less in stroke mice at 24 h when compared to sham-operated mice (Fig. 5B). There were detectable infarct and oedema volumes at 24 h post-stroke (20.79 mm^3^ and 16.42 mm^3^ respectively). There were no detectable infarcts or oedema in sham-operated mice (data not shown).

### Effect of 60 min occlusion on BALF cellularity and lung weight, structure and proinflammatory gene expression

**Figure 6:**
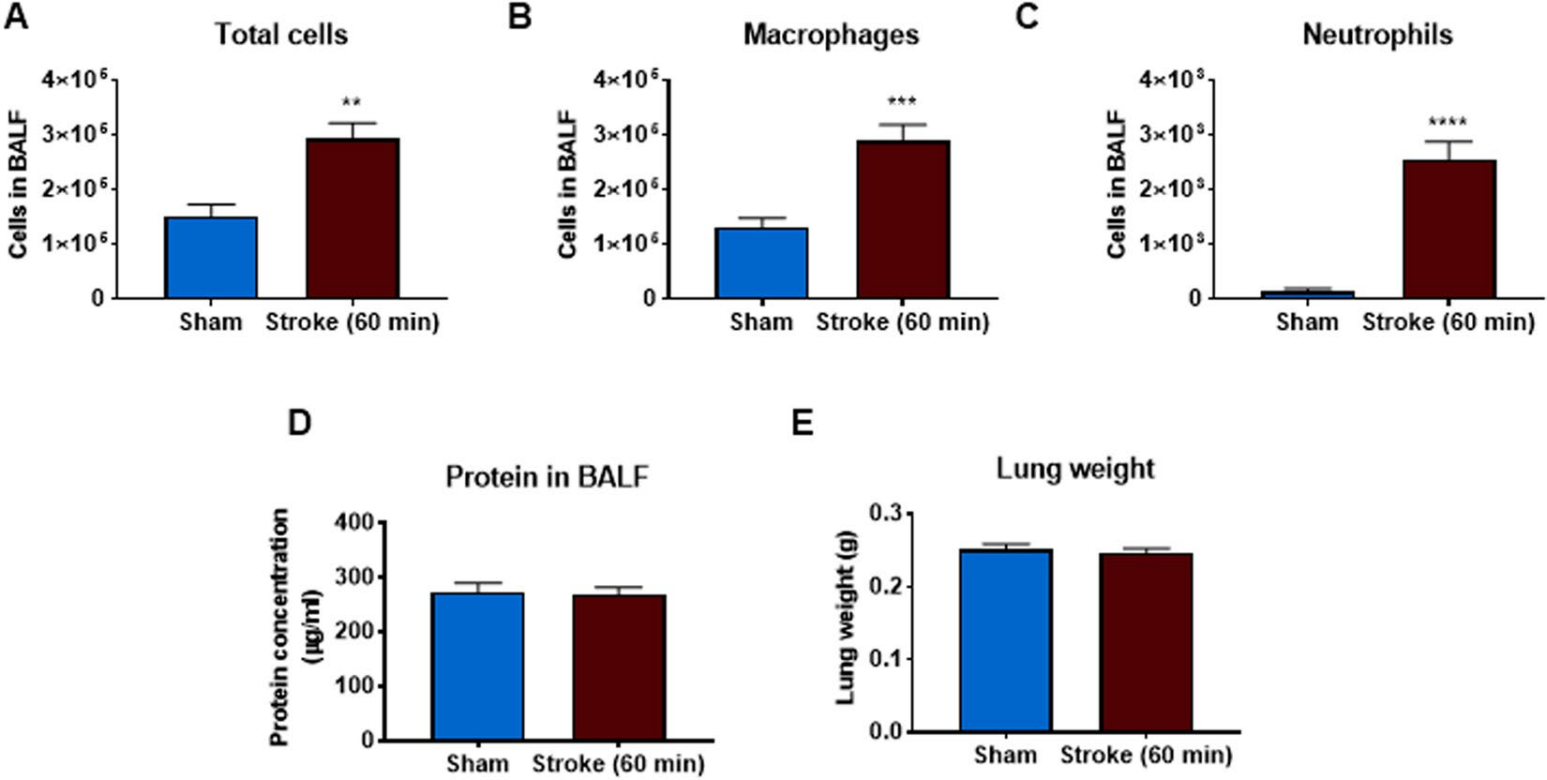
Effect of 60 min ischaemia on inflammatory cell counts in BALF, lung weight and BALF protein concentration. BALF cellularity is shown as (**A**) the total number of cells, (**B**) macrophages and (**C**) neutrophils. Protein concentration in BALF (**D**) and lung weight (**E**) data are also shown (n = 5–10). Data are expressed as mean ± SEM. Student’s t-test was performed to assess statistical significance (*P < 0.05 vs sham, **P < 0.005 vs sham, ***P < 0.0005 vs sham).

**Figure 7:**
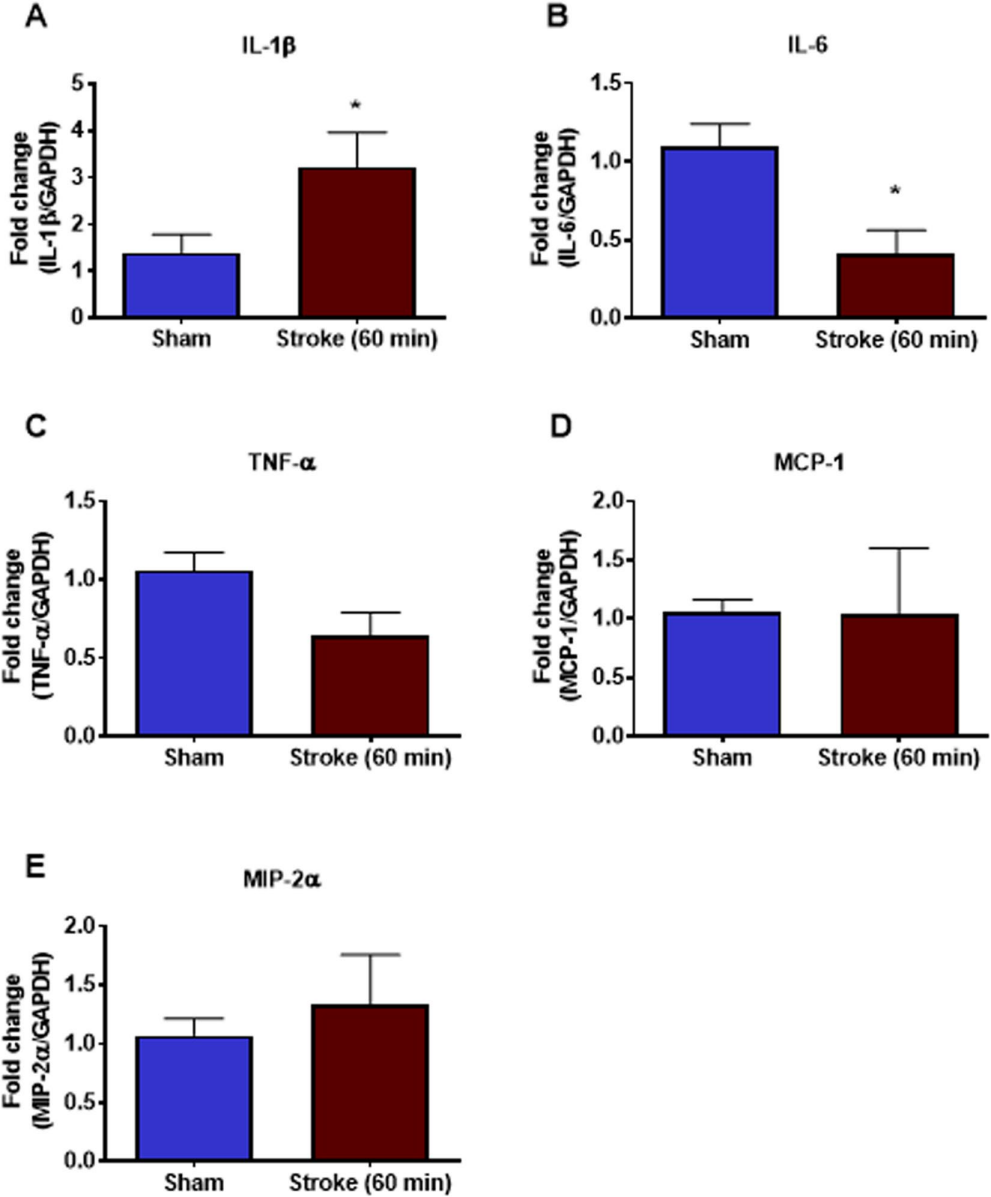
Effect of 60 min ischaemia on mRNA expression of proinflammatory cytokines and chemokines in whole lung tissue. Data are expressed as mean ± SEM. Student’s t-test was performed to assess statistical significance (*P < 0.05; n = 5–10).

**Figure 8:**
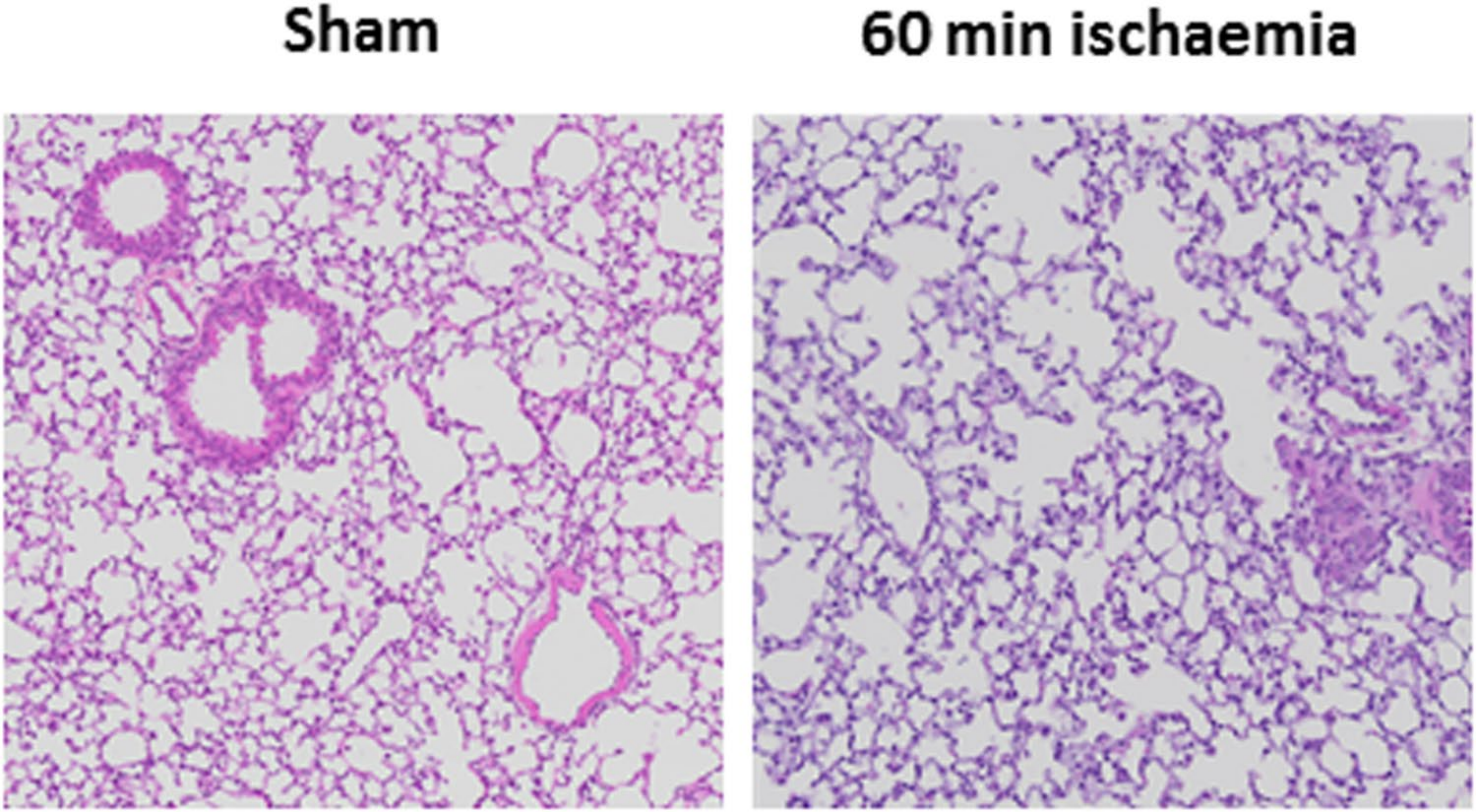
Hematoxylin and eosin stained lung tissue sections from mice 24 h post 60 min post stroke (60 min ischaemia) or sham surgery.

When mice were subjected to a 60 min occlusion period there was a significant increase in BALF total cells, macrophages and neutrophils when compared to sham-surgery mice (Fig. 6A–C). However, the 60 min occlusion did not affect lung weights or BALF protein content (Fig. 6D,E). Whole lung gene expression of IL-1β was significantly increased but levels of IL-6 were significantly decreased (Fig. 7A,B). However, the levels of TNF-α, MCP-1 and MIP-2α were not altered in stroke mice when compared to sham-surgery mice (Fig. 7C–E). Moreover, no evidence of lung injury was observed following the 60 min occlusion as assessed by histological examination of lung tissue (Fig. 8).

## Discussion

ALI is a clinical syndrome characterised by an acute inflammatory process in the lung tissue and airways, leading to an acute onset of severe hypoxemia. It is well known that brain injury is associated with ALI and ARDS, which is linked to mortality and leaves a high proportion of potential organ donors–those with fatal brain injury - unsuitable for lung transplants. The extent to which ALI occurs after ischemic stroke is not well established. This study aimed to determine whether experimental ischemic stroke causes ALI is in mice. We found that experimental ischemic stroke caused significant BALF inflammation (increase in macrophages and neutrophils) and an increase in whole-lung pro-inflammatory cytokines (IL-1β) but did not result in ALI and ARDS.

In this study, we used two occlusion periods of 50 min and 60 min to mimic a moderate and more severe stroke. It was predicted that the more severe stroke (i.e. 60 min occlusion period) would lead to a greater lung inflammatory response. In the 50 min occlusion experiments we found that the there was no significant increase in BALF inflammation at 6 and 72 hours post-stroke. However, there was a significant increase in the total number of macrophages 24 hours post stroke. Interestingly, in the more severe stroke, we found that there was a significant increase in the total cells, macrophages and neutrophils when assessed 24 hours after stroke. Therefore, it is clear that experimental ischemic stroke causes BALF inflammation.

The increase in neutrophils is in accord with findings that neutrophils are involved in the pathogenesis of ALI, causing lung endothelial damage which leads to pulmonary oedema^15–18^. Damage to the lung epithelial and endothelial cells in ALI allows for the movement of protein and fluid into the airways. However, despite the presence of BALF neutrophilia, experimental ischemic stroke did not cause pulmonary oedema, as assessed by lung weights or protein concentration in BALF. This was surprising given the significant BALF inflammation suggesting that lung epithelial and endothelia cells would have been damaged to allow oedema to occur. This would suggest that 60 min occlusion period still isn’t severe enough to see the full ALI/ARDS and that longer ischemic periods may be required. Thus, it would be worth trialling longer ischemic periods to determine their impact on lung inflammation, oedema and plasma leakage. Thus, it would be worth trialling longer ischemic periods to determine their impact on lung inflammation, oedema and plasma leakage. Based on the stroke literature we could use ischemic periods ranging from 60 and 120 minutes^25,31,32^.

While we were able to show increased BALF inflammation in stroke mice, we did not observe any increases in lung tissue inflammation as assessed by histology. This was surprising given the increased BALF inflammation and is in contrast to a recent study by Samary *et al*.^19^ who found that stroke mice had increased intra-alveolar oedema and increased macrophage counts when using transmission electron microscopy. It was also interesting that they did not see increased lung neutrophilia. However, it must be noted that Samary *et al*. used a focal ischemic model of stroke (thermo-coagulation of pial vessels over the right primary sensorimotor cortex) and rats, which may account for the observed differences.

We then went on to explore whether there was proinflammatory cytokine/chemokine expression in whole lung tissue as assessed by QPCR. Although no significant changes in pro-inflammatory cytokine (IL-1β, IL-6, TNF-α) and chemokine (MCP-1, MIP-2α) gene expression were observed at 6 h, 24 h and 72 h after the 50 min ischemic occlusion, IL-1β, IL-6 and MIP-2α expression tended to increase 6 h after stroke. However, changes in pro-inflammatory cytokine and chemokine gene expression were more obvious at 24 h after a longer (i.e. 60 min) occlusion, with a significant increase in IL-1β and decrease in IL-6. However, there were no differences in TNF-α, MCP-1 and MIP-2α. Of these pro-inflammatory mediators, IL-1β is known to be involved in the development of ALI/ARDS, and has been shown to be one of the most biologically active cytokines in ALI. IL-1β has been shown to stimulate the production of a number of cytokines^17^, and is thought to increase the permeability of lung epithelial and endothelial cells and contribute to the development of pulmonary oedema. Thus, gene expression analysis of pro-inflammatory cytokines in lung tissue indicated that lung inflammation is present after ischemic stroke. The recruitment and activation of neutrophils into the airways is a key feature of ALI in many disease contexts. In this study, however, no evidence of airway neutrophil infiltration was observed histologically after any length of ischaemia. Lung inflammation was present after experimental ischaemic stroke in this study, but not ALI.

## Conclusion

This study showed that ischemic stroke causes BALF inflammation and while there was increased proinflammatory gene expression in the whole lung this did not translate into histologically evident lung inflammation, oedema and lung injury which are the characteristic features of ALI. It is possible that occlusion times greater than 60 min are required to induce a more severe stroke for ALI to occur so further studies will need to be conducted to confirm this. Alternatively, it could be that ischemic stroke doesn’t cause ALI and ARDS in its more severe form. If this were the case, then this would mean that lungs from patient’s dying of ischemic stroke could be used for transplantation in people awaiting this life-saving therapy.

## Data Availability

All data generated or analysed during this study are included in this published article.
